# Divergent volatilomes between wild and cultivated strawberries: MYB transcription factors underlie flavor differences

**DOI:** 10.1016/j.fochms.2025.100323

**Published:** 2025-11-04

**Authors:** Mingzheng Duan, Kangjian Song, Ting Jiang, Xiaoting Fu, Huaming Lei, Jieming Feng, Congjing Chen, Xiande Duan, Shunqiang Yang, Muhammad Junaid Rao

**Affiliations:** a Advanced Institute of Ecological Agriculture and Biodiversity on the Yunnan-Guizhou Plateau, College of Agronomy and Life Sciences, Zhaotong University, Zhaotong 657000, China; b State Key Laboratory for Development and Utilization of Forest Food Resources, Zhejiang A&F University, Hangzhou 311300, China

**Keywords:** Strawberry volatilome, MYB transcription factors, Aroma biosynthesis genes, GC–MS metabolomics

## Abstract

Strawberry (*Fragaria* spp.) flavor is shaped by a complex interplay of volatile organic compounds (VOCs) and their regulation by MYB transcription factors, yet the genetic and biochemical divergence between wild and cultivated varieties remains poorly understood. This study employed integrated transcriptomic and metabolomic analyses to compare a wild strawberry (*F. nilgerrensis*) with cultivated (*F. × ananassa*) varieties. We identified distinct volatile signatures: cultivated fruits were enriched in fruity-floral volatiles such as nerolidol, methyl anthranilate, and various esters, whereas the wild genotype accumulated stress-associated metabolites like geranyl acetate and 2,3-dihydroxy-benzoic acid. Expression profiling revealed key MYB transcription factors (e.g., FxaYL_542g0723070, FxaYL_512g0659290) whose abundance strongly correlated with these divergent phenylpropanoid and terpenoid volatile profiles. These findings reveal a metabolic trade-off in cultivated strawberries; whereby sensory traits are enhanced relative to defense mechanisms. This comparison provides molecular targets for breeding strawberries with enhanced flavor and resilience. This work advances our understanding of strawberry aroma biochemistry and offers a strategic roadmap for developing cultivars with superior flavor and resilience.

## Highlights

• Cultivated strawberries accumulate fruity-floral volatiles; wild relatives retain defense metabolites.
• MYB transcription factors are strongly correlated with key aroma compounds like nerolidol and methyl anthranilate.
• Distinct MYB gene expression patterns underpin the flavor divergence between wild and cultivated varieties.
• This study provides candidate MYB genes for breeding flavor-rich, resilient strawberries.

## 1 Introduction

Strawberries (*Fragaria* spp.) rank among the world's most beloved and economically important berry crops, valued for their vibrant color, succulent texture, and complex flavor profile that balances sweetness with subtle acidity (Aharoni et al., 2004). Globally, *F. × ananassa* dominating commercial markets and it's the most widely consumed fruits, due to its large fruit size and superior sensory attributes (Ramya et al., 2017). The fruit's sensory appeal stems from a dynamic blend of sugars, organic acids, and volatile organic compounds (VOCs), including esters, terpenes, furanones, and phenolic derivatives (Xu et al., 2025). Beyond taste, strawberries are rich in bioactive phytochemicals (anthocyanins, ellagitannins, and ascorbic acid), which underlie their nutritional appeal and antioxidant capacity (Rao, Duan, et al., 2025; Rao, Wang, et al., 2025; Zheng et al., 2025). Among berries, strawberries are unique in their rapid postharvest flavor deterioration, making the biochemical understanding of their aroma pathways critical for breeding longer-lasting, high-quality varieties (Pott et al., 2020). The genetic basis of these traits, however, remains partially deciphered, particularly regarding the genetic changes that underlie the divergent secondary metabolite landscape between wild and cultivated strawberries.

The divergence between wild and cultivated strawberries provides a unique opportunity to explore the genetic basis of fruit quality (Aharoni et al., 2004). Wild varieties, such as *Fragaria nilgerrensis*, often exhibit starkly different metabolic profiles compared to cultivated hybrids (*F. × ananassa*), likely reflecting trade-offs between palatability and ecological adaptation (Hu et al., 2022; Wang et al., 2024). For instance, domesticated strawberries may prioritize sweeter, fruitier aromas, while wild types retain compounds linked to stress resistance or herbivore deterrence (Chamorro et al., 2025). Understanding these differences could inform breeding strategies to enhance flavor without compromising resilience. Recent advances in metabolomics and transcriptomics offer powerful tools to dissect these pathways, revealing how key regulatory genes, such as MYB transcription factors, influence the synthesis of flavor-associated metabolites.

MYB transcription factors represent one of the largest and most versatile gene families in plants, playing pivotal roles in regulating secondary metabolite pathways that directly influence fruit quality, color, and aroma (Dubos et al., 2010). They orchestrate the biosynthesis of flavonoids, anthocyanins, and volatile organic compounds (VOCs) by modulating key enzymes in phenylpropanoid, terpenoid, and fatty acid-derived pathways across diverse species (Gu et al., 2022). For instance, in grapes (*Vitis vinifera*), MYB activators such as VvMYB5a and VvMYBPA1 control the accumulation of proanthocyanidins and anthocyanins (Lafferty et al., 2022; Ramya et al., 2017), while in tomatoes (*Solanum lycopersicum*), SlMYB12 regulates flavonols and hydroxycinnamic acids, impacting flavor and antioxidant capacity (Cao et al., 2024). This MYB-dependent regulation extends to the monoterpenes that define aroma in various crops (Jian et al., 2019; Lv et al., 2022; Ramya et al., 2017), underscoring the family's broad importance in shaping fruit sensory attributes.

In strawberries (*Fragaria* spp.), the regulatory roles of specific MYB TFs are increasingly being elucidated. Well-characterized examples include FaMYB10, a master regulator of anthocyanin biosynthesis (Yue et al., 2023), and FaEOBII and FaMYB63, which directly influences aroma by controlling the synthesis of the volatile eugenol (Medina-Puche et al., 2015; Wang, Shi, et al., 2022; Wang, Zhou, et al., 2022). A novel R2R3-MYB gene *FaMYB5* has been implicated in the anthocyanin and proanthocyanidin pathways and involved in regulating citric acid metabolism (Jiang et al., 2023; Liu et al., 2022), indirectly and directly shaping fruit chemistry and texture. Furthermore, the R2R3-MYB repressor FaMYB44.2 has been identified as a key regulator of sugar and acid metabolism, whose antagonistic interaction with FaMYB10 provides an elegant model for the coordinated control of multiple ripening traits (Wei et al., 2018). While these studies provide crucial insights, they focus on a limited number of characterized MYBs. Consequently, the extent to which MYB divergence between wild and cultivated strawberries drives differences in volatile profiles remains poorly understood (Chamorro et al., 2025). This study focuses on elucidating these regulatory mechanisms by profiling MYB expression across diverse strawberry genotypes and correlating it with VOC signatures. By identifying MYB genes that co-occur with elevated levels of desirable aroma compounds, we aim to uncover breeding targets for enhancing flavor complexity.

In this study, we employ an integrated metabolomic and transcriptomic approach to unravel the complex interplay between genetic regulation and volatile compound biosynthesis in strawberries. Our primary objectives includes: (1) to characterize and compare the volatile organic compound (VOC) profiles of wild (*Fragaria nilgerrensis* ‘HM’) and cultivated (*F. × ananassa* ‘Red Face 99’, ‘Fenyu’, and ‘Danxue’) strawberry varieties using advanced GC–MS techniques; (2) to identify differential expression patterns of MYB transcription factors, known regulators of secondary metabolite pathways, through RNA sequencing; and (3) to establish meaningful correlations between specific MYB genes and key aroma compounds that define strawberry flavor. By bridging these molecular and biochemical analyses, we aim to uncover how breeding and selection have reshaped the fruit's sensory chemistry, potentially at the expense of stress-related metabolites retained in wild genotypes. These investigations will not only advance our fundamental understanding of strawberry flavor biochemistry but also provide actionable insights for targeted breeding programs. The findings could identify candidate MYB regulators for strategic manipulation aimed at enhancing desirable flavor traits while preserving or reintroducing valuable wild-type characteristics, such as disease resistance.

## 2 Material and methods

### 2.1 Sample collection and processing

Fresh strawberry fruits from four distinct cultivars were analyzed: ‘Red Face 99’ (*Fragaria × ananassa* ‘RF’, exhibiting red pigmentation), ‘Fenyu’ (*F. × ananassa* ‘FY’, displaying pink coloration), ‘Danxue’ (*F. × ananassa* ‘DX’, characterized by white appearance), and wild yellow-haired strawberry (*Fragaria nilgerrensis* ‘HM’, presenting white phenotype). Plants were grown under controlled greenhouse conditions. Fruits were harvested at the fully ripe stage (March 2024), as determined by full surface coloration specific to each cultivar. For each variety, a biological replication consisted of a pool of fruits from five plants grown in a single pot. Three independent biological replicates (pots) were sampled per variety. Selection criteria included consistent maturity stage and absence of physical defects.

Following peduncle removal, specimens underwent gentle washing with deionized water to remove external debris, followed by air-drying on sterilized filter paper. Using aseptic scalpel techniques, fruits were sectioned into uniform fragments (approximately 0.5 cm^3^). Samples were systematically randomized, enclosed in aluminum foil, and flash-frozen by immersion in liquid nitrogen for 2–5 min. Each frozen biological sample was then finely ground to a homogeneous powder, and aliquoted into three technical replicates for subsequent metabolomic and transcriptomic analysis. Subsequently, processed samples were maintained at −80 °C until evaluation.

### 2.2 RNA sequencing and transcriptome profiling of strawberry fruits

Total RNA was isolated from strawberry fruit samples utilizing the Invitrogen Trizol reagent system (Carlsbad, CA, USA). RNA quality assessment, library construction, and high-throughput sequencing services were performed by Berry Hekang Biotech (Beijing, China). Sequencing libraries were prepared according to standard protocols using the NEBNext UltraTM RNA Library Prep Kit for Illumina sequencing (NEB, Ipswich, MA, USA). High-quality paired-end sequences were generated on the Illumina HiSeq 2500 sequencing platform following quality filtration procedures. The analysis employed the *Fragaria vesca* v6 reference genome assembly (Fragariavescav6genome.fasta) obtained from http://eplantftp.njau.edu.cn/Fragaria/F._vesca/F._vesca_v6.0/, with corresponding structural annotations (Fragariavescav6genome.gff). Reads were aligned to high-quality *F. vesca* v6 reference genome because it provides a well-annotated and stable framework for identifying homologous genes across *Fragaria* species, a common practice for comparative transcriptomics in this genus due to the complexity of the octoploid genome. Quality-filtered reads were aligned to the reference genome using HISAT2 alignment software.

Functional annotation of transcripts was performed through integration of multiple bioinformatics databases including Kyoto Encyclopedia of Genes and Genomes Ortholog groups (KO), Protein family classifications (Pfam), Gene Ontology terms (GO), NCBI nucleotide database (Nt), Swiss-Prot curated protein sequences, Clusters of Orthologous Groups classifications (KOG/COG), and NCBI non-redundant protein database (Nr). Sequence preprocessing involved adapter trimming using Cutadapt software, with RNA-seq data quality evaluation conducted through FastQC analysis. Differential gene expression analysis was performed using Cuffdiff, applying stringent criteria of |Log₂(fold change)| ≥ 1.0 and adjusted *p*-value <0.05 for statistical significance (MYB gene expression-Supplementary Table S1). Gene Ontology enrichment testing of differentially expressed transcripts was conducted using the GOseq R package, which implements the Wallenius non-central hypergeometric distribution to correct for gene length bias. Pathway enrichment analysis was performed using KOBAS software to identify significantly overrepresented KEGG biological pathways among differentially expressed genes (Mao et al., 2005).

### 2.3 Analysis of volatile compounds using gas chromatography-mass spectrometry

Volatile metabolite profiling of strawberry fruit samples was conducted through solid-phase microextraction (SPME) coupled with gas chromatography–mass spectrometry. Fresh fruit tissue (500 mg) was transferred to sealed headspace vials containing sodium chloride solution and a designated internal standard, then equilibrated at 60 °C for 5 min. Volatile compounds were extracted using a 120 μm DVB/CWR/PDMS SPME Arrow fiber exposed to the sample headspace for 15 min at 60 °C, subsequently desorbed at 250 °C for 5 min in the gas chromatograph injector port (Agilent Model 8890) (Agilent Technologies Co., Ltd., Shanghai, China).

Chromatographic separation was achieved using a DB-5MS fused silica capillary column (30 m length × 0.25 mm internal diameter × 0.25 μm film thickness) with 5 % phenyl-polymethylsiloxane (Anhui ZINCA Silicone Technologies Co., Ltd., Anhui, China) stationary phase. Helium served as the carrier gas at a constant flow rate of 1.2 mL/min. The injection port temperature was maintained at 250 °C throughout the analysis. The column oven temperature program initiated at 40 °C with a 3.5-min hold, followed by a temperature ramp to 280 °C and a final isothermal period of 5 min.

Mass spectrometric detection employed electron impact ionization at 70 eV operating in selected ion monitoring (SIM) mode. The ion source and quadrupole analyzer temperatures were set to 230 °C and 150 °C, respectively. Data acquisition and processing were performed using MassHunter analytical software for both qualitative identification and quantitative determination (Yuan et al., 2024). Detailed mass spectrometric parameters for individual compounds are presented in Supplementary Table S2.

### 2.4 Statistical analysis

Integrated multivariate analytical frameworks were implemented to examine both metabolomic and transcriptomic datasets, facilitating effective discrimination of metabolic compounds and their corresponding gene expression patterns. The analytical strategy was designed to enhance inter-group variability while maintaining intra-group consistency. Dataset normalization procedures were applied to all data matrices prior to hierarchical clustering analysis (HCA) of metabolomic profiles, following established methodological frameworks (Mu et al., 2024). Both HCA and principal component analysis (PCA) were executed according to validated protocols described in previous studies (Chanana et al., 2020). Only metabolites with a |log2FC| ≥ 1.5 and *p*-value <0.05 were included in HCA and PCA analysis.

Variable Importance in Projection (VIP) values were determined using orthogonal partial least squares-discriminant analysis implemented through the MetaboAnalystR computational package within the R statistical environment (Chong & Xia, 2018). Metabolites demonstrating VIP values greater than 1.0 were classified as key discriminatory features between experimental groups (Thévenot et al., 2015). Significantly altered metabolites were identified using stringent dual-threshold criteria: statistical significance assessed through Student's *t*-test with false discovery rate adjustment (*P* < 0.05), combined with biological significance determined by fold-change thresholds (|log2FC| ≥ 1, corresponding to FC ≥ 2 or ≤ 0.5) (Fraga et al., 2010). Additional graphical elements were prepared using Microsoft Excel (Redmond, WA, USA). Comparative analysis of differentially expressed metabolite overlap across sample groups was performed through Venn diagram construction using the EVenn web-based platform (http://www.ehbio.com/test/venn/#/) (Mu et al., 2024).

## 3 Results

### 3.1 Clustering analysis of MYB genes in strawberry varieties

**Fig. 1:**
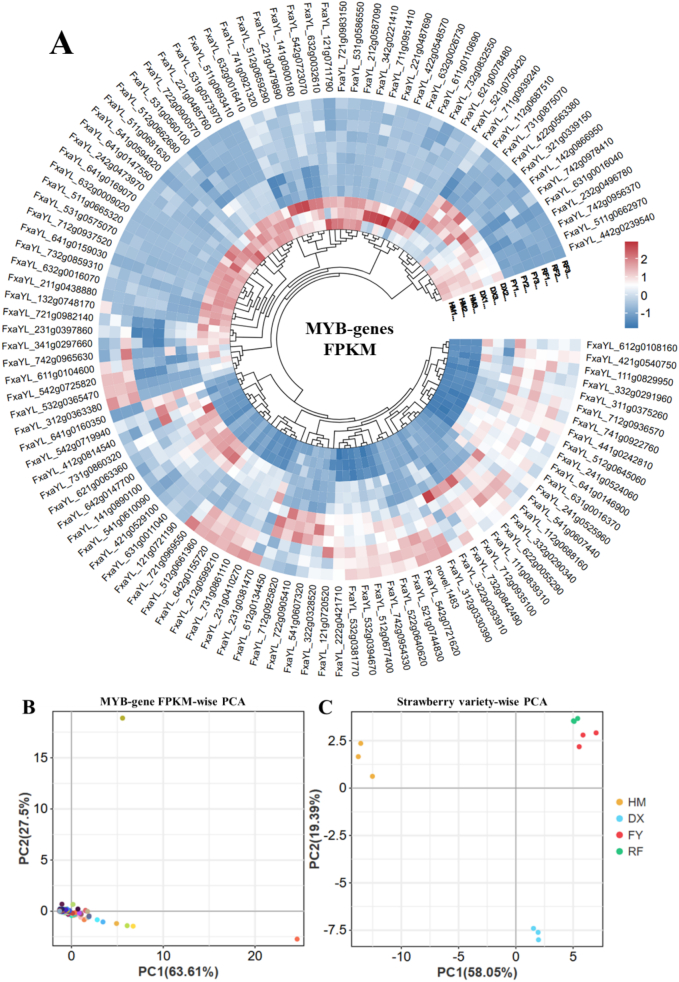
Hierarchical clustering analysis and principal component analysis of MYB gene expression across four strawberry varieties reveal distinct regulatory signatures. A: HCA of differentially expressed MYB Genes (|Log₂(fold change)| ≥ 1 and *p*-value <0.05), B: MYB Gene-wise PCA, PCA of individual MYB genes displays their expression divergence along PC1 and PC2, C: Strawberry Variety-wise PCA, PCA of MYB expression profiles across varieties underscores the transcriptional gulf between wild (HM) and cultivated (DX, FY, RF) strawberries.

The hierarchical clustering analysis (HCA) of MYB transcription factors across four strawberry varieties (wild HM and cultivated DX, FY, and RF) reveal distinct patterns of gene expression patterns, likely influencing key flavor and aroma compounds. MYB genes are crucial regulators of phenylpropanoid and terpenoid pathways, which contribute to the synthesis of volatile organic compounds (VOCs) such as esters, terpenes, and phenolics. The dendrogram shows a clear separation between some gene clusters, suggesting differential regulation of MYB genes between wild and cultivated varieties. The wild yellow-haired strawberry (HM) shows low or absent expression in many MYB genes such as FxaYL_642g0155720, FxaYL_112g0688160, FxaYL_231g0410270, FxaYL_612g0108160, and FxaYL_121g0721190 compared to cultivated varieties. The heightened expression of these genes in cultivated varieties correlates with improved fruit quality traits. Cultivated strawberries (DX, RF and FY varieties) display significantly higher MYB expression for genes like FxaYL_642g0147700 and FxaYL_712g0936570 whereas HM showed least/zero expression of these genes (Fig. 1A). Interestingly, some MYBs (e.g., FxaYL_632g0009020) are exclusively expressed in HM, hinting at wild-specific metabolic adaptations, possibly linked to stress resistance or less palatable secondary metabolites (Fig. 1A).

The pink strawberry (FY) shows unique MYB activation patterns, such as high expression of FxaYL_112g0688160 and FxaYL_732g0842490 whereas HM showed least expression of these genes, which may correlate with its distinct volatile profile. Conversely, HM retains some highly expressed MYBs (e.g., FxaYL_221g0487690 that are suppressed in cultivated varieties, suggesting a trade-off between flavor and wild-type defense mechanisms (Fig. 1A). These patterns highlight MYBs as key targets for precision breeding—enhancing specific genes could further refine flavor in commercial varieties while reintroducing wild-type MYBs might improve stress resilience without compromising taste. Overall, the HCA provides insights into the evolutionary and functional divergence of MYB genes in strawberries, offering a foundation for future genetic studies.

The PCA of MYB gene expression in strawberries reveals distinct regulatory patterns, with PC1 explaining 63.61 % of the variance and PC2 capturing 27.5 %, indicating strong transcriptional divergence across genes (Fig. 1B). The wide dispersion of MYB genes along PC1 suggests polarized expression profiles, where some genes are highly active in cultivated varieties (likely driving flavor and aroma pathways like terpenoid and phenylpropanoid biosynthesis), while others are suppressed or wild-type specific. For instance, genes clustered toward higher PC1 values may suggest a role in fruity ester production, aligning with cultivated strawberries' enhanced aroma, whereas those at lower PC1 values could govern defensive or stress-responsive traits retained in wild types (Fig. 1B). Along PC2, tightly grouped genes may represent core transcriptional networks conserved across varieties, while outliers could signify unique regulators of key volatiles.

The PCA of MYB gene expression across strawberry varieties reveals striking transcriptional differences associated with breeding and selection, with PC1 (58.05 % variance) and PC2 (19.39 % variance) clearly separating wild (HM) from cultivated (DX, FY, RF) types (Fig. 1C). The wild variety HM clusters are distinctly at one extreme of PC1, suggesting a unique MYB regulatory network, likely tuned for stress resistance and ecological adaptation, with lower expression of genes associated with flavor-enhancing pathways. In contrast, cultivated varieties group together but exhibit subtle divergences: FY (pink) and RF (red) strawberries show closer proximity, possibly reflecting shared MYB-associated traits like elevated terpenoid or ester production, while DX may represent an intermediate profile. These insights could guide breeding strategies to reintroduce resilience-linked MYB genes without compromising flavor.

### 3.2 Differential metabolites (VIP > 1.0 and *p* < 0.05) in strawberry varieties

**Fig. 2:**
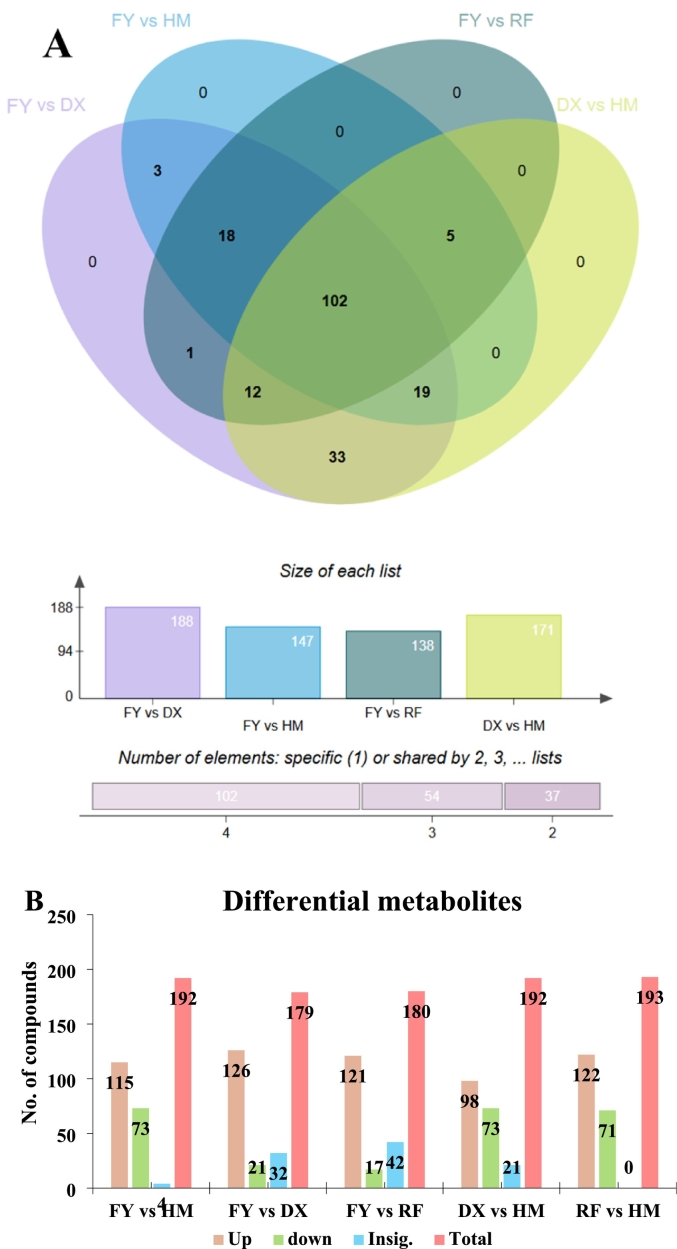
Shared and unique volatile compounds among strawberry varieties. A: Venn diagram illustrating the overlap of volatile organic compounds detected in four strawberry genotypes (wild HM and cultivated FY, DX, RF), B: Differential volatile compound patterns between different comparison groups of strawberry varieties, all metabolites were significantly up-regulated and down-regulated (VIP > 1.0 and *p* < 0.05; OPLS-DA plots are represented in Supplementary fig. S1).

A total of 193 volatile organic compounds (VOCs) were detected across all strawberry samples using GC–MS analysis (Supplementary Table S3). Subsequent analyses focused on the compounds that were significantly altered between groups, identified using PLS-DA with thresholds of VIP > 1.0 and *p* < 0.05. The Venn diagram analysis (Fig. 2A) highlights both unique and overlapping volatile compounds among four strawberry varieties (FY, HM, RF, and DX). The FY vs DX group showed 188 compounds that significantly altered followed by DX vs HM 171, FY vs HM 147 and the lowest number of altered volatile compounds were exhibited in FY vs RF group. The intersection of all four comparison groups of strawberry varieties revealed commonly shared of 102 compounds (Fig. 2A). This demonstrates substantial diversification in volatile compound composition among varieties. These findings provide valuable insights for flavor-oriented breeding programs, emphasizing the need to balance novelty with the preservation of key aroma compounds.

The differential metabolites analysis (VIP > 1.0 and p < 0.05) demonstrates distinct VOCs differences between strawberry varieties, with RF and FY showing the most pronounced differential VOCs when compared to other genotypes. Notably, RF vs HM (122 upregulated, 72 downregulated) and FY vs HM exhibited 115 upregulated and 73 downregulated metabolites, suggesting substantial metabolic divergence between this cultivated variety and the wild HM (Fig. 2B). In contrast, comparisons between FY and other cultivated varieties (DX and RF) also revealed fewer differentially VOCs (FY vs DX: 126 up, 21 down; FY vs RF: 121 up, 17 down), indicating distinct VOCs relationships among cultivated genotypes (Fig. 2B). The DX vs HM comparison showed intermediate differences (98 up, 73 down), highlighting that the cultivated genotype retains a distinct volatile profile from its wild relative (Fig. 2B).

These results suggest that FY has undergone significant metabolic reprogramming during breeding, potentially contributing to its unique phenotypic characteristics. The high number of insignificant genes in FY vs DX (32) and FY vs RF (42) comparisons imply conserved regulatory networks among cultivated varieties, while the limited insignificant genes in FY vs HM (4) underscores the wild-cultivated divide (Fig. 2B). These metabolic patterns may reflect selection pressures for traits such as fruit quality or stress tolerance, providing valuable targets for future molecular breeding efforts aimed at enhancing desirable strawberry characteristics.

### 3.3 GC–MS metabolites clustering and grouping of wild and cultivated strawberries

**Fig. 3:**
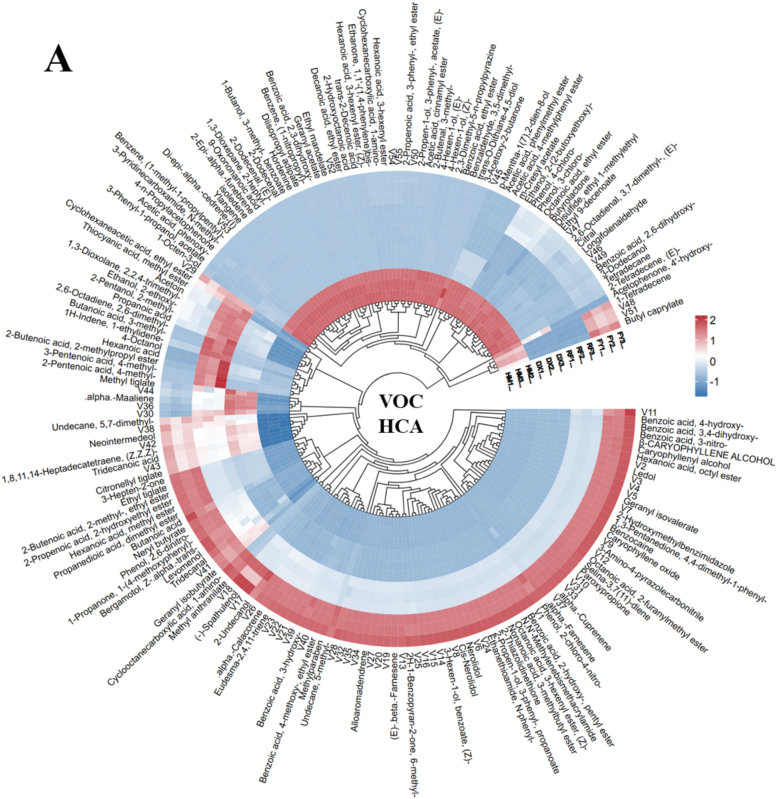

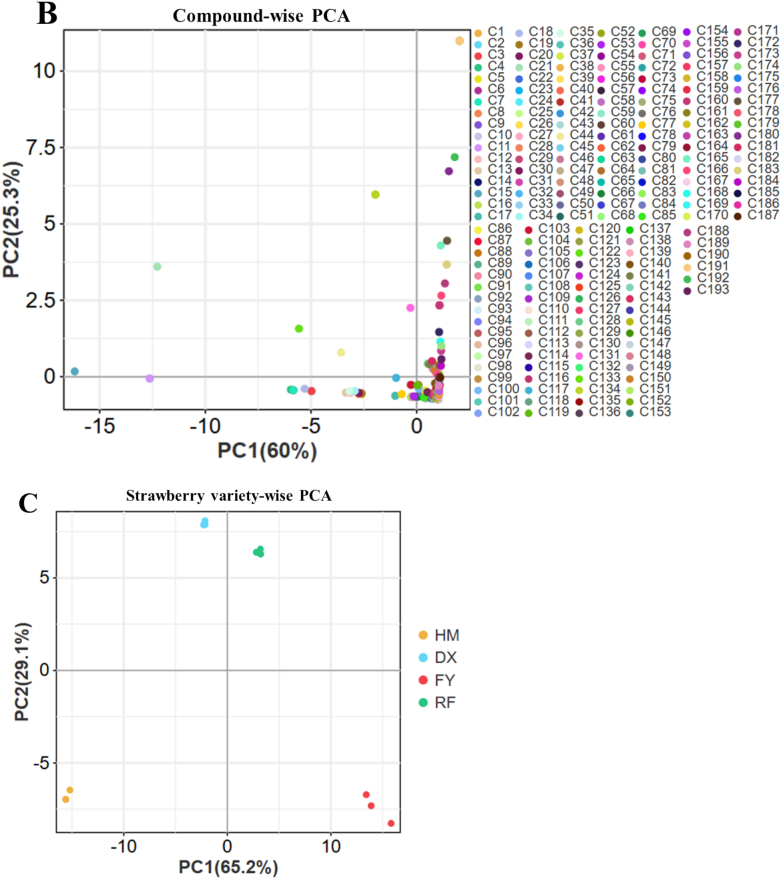
Hierarchical clustering analysis and principal component analysis of volatile organic compounds (VOCs) across wild (HM) and cultivated (DX, FY, RF) strawberry varieties. Only metabolites with a |log2FC| ≥ 1.5 and p-value <0.05 were included in this analysis. A: HCA of VOC among strawberries (V1-V55 number are consistent with individual compound represented in supplementary Table S4), B: Compound-wise PCA: Principal component analysis of 193 VOCs (C1–C193 number are consistent with individual compound represented in supplementary Table S4), illustrating how chemical diversity drives metabolic divergence, C: Variety-wise PCA: PCA of strawberry varieties based on VOC profiles shows HM's distinct separation from DX, FY, and RF.

Hierarchical cluster analysis of the GC–MS data revealed distinct clustering patterns among the four strawberry varieties, with the wild yellow-haired strawberry (HM) showing a clear separation from the cultivated varieties, particularly the pink strawberry (FY) (Fig. 3A). The heatmap and dendrogram highlighted a stark contrast in volatile compound profiles, with FY exhibiting significantly higher abundances of key metabolites such as neryl butyrate, butanoic acid, propanedioic acid dimethyl ester, hexanoic acid methyl ester, 2-propenoic acid 2-hydroxyethyl ester, 2-butenoic acid 2-methyl- ethyl ester, and ethyl tiglate etc., compared to HM (Fig. 3A). Additionally, compounds such as nerolidol, geranyl isovalerate, and benzoic acid derivatives were also markedly elevated in FY than HM, whereas the HM showed high abundance of geranyl acetate, 2,3-dihydroxy-benzoic acid, ethyl mandelate, hordenine, diisopropyl adipate, etc., then FY, suggesting a strong divergence in aroma and flavor biosynthesis between the wild and cultivated strawberries (Fig. 3A). The HCA clearly differentiates cultivated varieties from wild (HM), highlighting how breeding and selection are linked to profound changes in volatile organic compound accumulation (Fig. 3A). FY's enrichment in fruity, floral, and sweet-associated metabolites aligns with its commercial appeal, while HM's profile reflects a more defensive, possibly less palatable, wild adaptation.

The PCA plot of volatile organic compounds (VOCs) in strawberries reveals clear metabolic distinctions between samples, with PC1 explaining 60 % of the total variance, a strong indication that the dominant chemical differences are captured along this axis (Fig. 3B). The wide spread of compounds (C1–C193) along PC1 suggests significant variation in VOC profiles, likely due to evolutionary divergence between wild and cultivated strawberries or varying biosynthetic pathways (Fig. 3B). Cultivated varieties might group on one end with higher sweet/floral volatiles (e.g., methyl anthranilate), while wild types cluster on the other with defensive compounds (e.g., benzoic acids). The tight clustering of some compounds (e.g., near −5) suggests shared metabolic regulation, possibly co-expressed pathways like terpenoid biosynthesis. Meanwhile, outliers may represent unique signatures of specific cultivars (Fig. 3B). Overall, the PCA highlights that the cultivated strawberries prioritize pleasant VOCs, while wild types retain metabolites for ecological resilience. This insight could guide breeding for balanced flavor and stress tolerance.

The PCA analysis of variety-wise VOCs reveals distinct signatures, with PC1 (65.2 % variance) and PC2 (29.1 % variance) capturing most differences (Fig. 3C). The clear separation of wild strawberry (HM) from cultivated varieties (DX, FY, RF) along PC1 highlights a fundamental divergence in VOC profiles, consistent with the distinct metabolic priorities of wild and cultivated types. Cultivated varieties cluster closer together, suggesting shared metabolic traits—such as elevated fruity esters or floral terpenes—bred for consumer appeal. In contrast, HM's distinct positioning indicates a unique VOC composition, potentially richer in defensive or herbivore-deterrent compounds. Along PC2, finer variations emerge among cultivated types, possibly differentiating their sensory attributes (e.g., FY's pink strawberries may show higher methyl anthranilate). The widespread along both axes underscores the genetic and biochemical diversity harnessed during strawberry breeding. Such insights could guide future breeding to reintroduce wild traits, like stress resistance, without compromising flavor.

### 3.4 Variations in key volatile compounds among strawberry genotypes

**Fig. 4:**
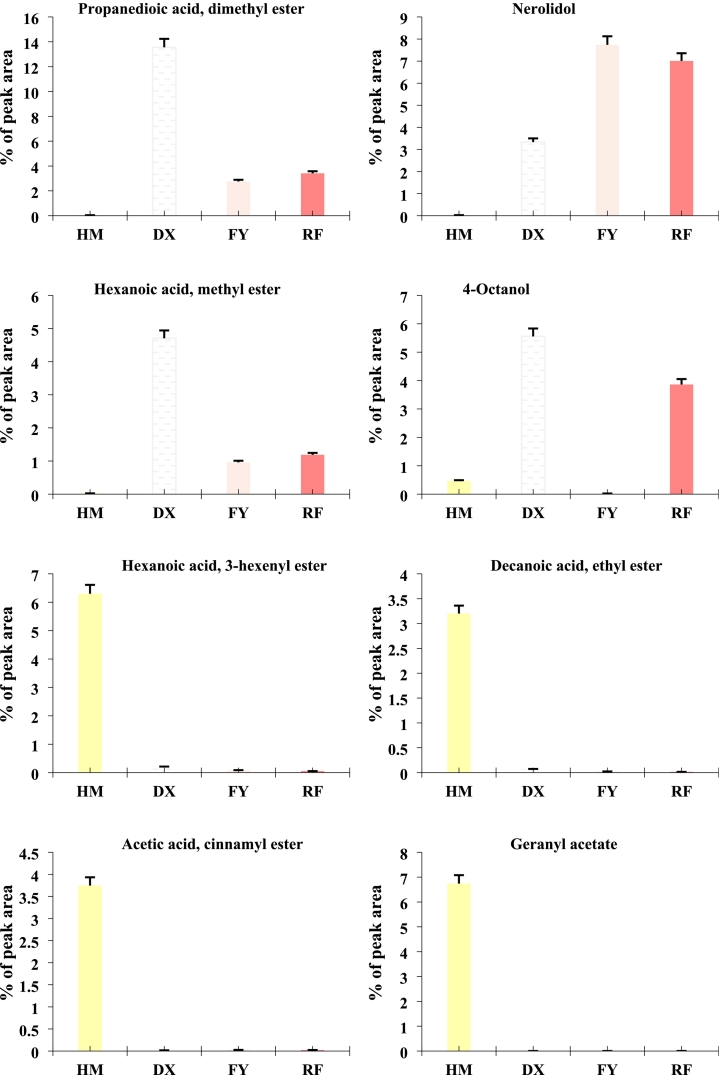
Relative abundance (% peak area) of key volatile compounds in four strawberry varieties (wild HM and cultivated DX, FY, RF). Only metabolites with a |log2FC| ≥ 1.5 and p-value <0.05 were included in this analysis. Data represents mean values, with error bars indicating standard deviation (*n* = 3).

The analysis of key volatile compounds in four strawberry varieties revealed distinct profiles in their peak area percentages, highlighting key differences in aroma and flavor potential (Fig. 4). Propanedioic acid, dimethyl ester and nerolidol were detected across all varieties, however, their relative abundances varied, with cultivated varieties (DX, FY, RF) often exhibiting higher or more consistent levels compared to the wild HM, possibly reflecting selective breeding for desirable aroma traits (Fig. 4). Notably, nerolidol, a sesquiterpene associated with floral and woody notes, showed marked variability (high in FY followed by RF and DX, lowest in HM), which may influence the sensory profiles of these varieties. Hexanoic acid, methyl ester, a contributor to fruity and sweet aromas, displayed significant differences among the varieties (Fig. 4). Cultivated varieties, particularly DX and FY, exhibited higher peak areas, indicating stronger emission of this ester, which aligns with their enhanced fruity flavor (Fig. 4). In contrast, 4-octanol, a less common volatile, was detected at trace levels, with no clear dominance in any variety except DX, suggesting it may play a minor role in aroma differentiation. These findings underscore the divergence in volatile compound composition between wild and cultivated strawberries, with cultivated types favoring compounds associated with sweeter, more complex aromas.

The hexanoic acid, 3-hexenyl ester and decanoic acid, ethyl ester displayed striking differences among varieties (Fig. 4). HM dominated with a peak area of 6.30 % and 3.2 % respectively, while cultivated varieties (DX, FY, RF) showed minimal levels (0.05–0.20 %) (Fig. 4). This sharp contrast implies that modern breeding may have inadvertently reduced this compound's abundance, possibly due to selection for other flavor traits or yield-related characteristics (Fig. 4). Additionally, acetic acid, cinnamyl ester and geranyl acetate, both associated with floral and fruity notes, were detected across all varieties but exhibited variable abundances (Fig. 4). The column graph illustrates that HM consistently shows a higher percentage of peak areas for both compounds compared to the cultivated varieties (Fig. 4). This suggests that the wild genotype may retain stronger floral aromatic characteristics, which may have been reduced during selection for other agronomic traits. Geranyl acetate, a monoterpene ester linked to citrus and rose-like scents, was particularly prominent in HM, hinting at its potential role in the unique aroma profile of wild strawberries (Fig. 4). These findings highlight the importance of conserving genetic diversity in breeding programs aimed at enhancing sensory quality in strawberries.

### 3.5 Metabolic shaping of strawberry aroma: upregulated and downregulated volatiles

The comparison between the cultivated strawberry variety ‘Fenyu’ (FY) and the wild ‘HM’ genotype revealed a significant upregulation of key volatile compounds. Differential metabolites were identified using PLS-DA analysis and considered significant based on a VIP score > 1.0 and a *p*-value <0.05. All compounds meeting these criteria are listed in Table 1 (up-regulated) and Table 2 (down-regulated), ordered by their log2 fold change. The most dramatic increase was observed for benzenemethanol acetate, an ester with a log2 fold change (log2FC) of 10.67, indicating an over 1600-fold enrichment in FY compared to HM (Table 1). Similarly, terpenoids such as ledol and caryophyllene oxide, known for their woody and spicy aromatic notes, exhibited log2FC values exceeding 10, suggesting that breeding and selection have strongly favored the accumulation of these compounds (Table 1). Notably, ester (Geranyl isovalerate, log2FC 10.29) and terpenoid (nerolidol, log2FC 10.12), both linked to fruity and floral scents, were among the most significantly elevated, supporting the hypothesis that modern breeding has enhanced volatile pathways responsible for consumer appeal (Table 1). The consistency in high log2FC values (ranging from 9.74 to 10.67) across multiple compound classes underscores a systemic shift in secondary metabolism, suggesting a potential role for human selection in shaping the flavor profile of cultivated varieties. These results provide critical targets for future breeding strategies, suggesting that further optimization of these pathways could refine aroma complexity while potentially reintroducing beneficial wild-type traits for stress resilience.

**Table 1:** All significantly up-regulated volatile compounds in ‘Fenyu’ (FY) compared to wild ‘HM’ strawberries, identified by PLS-DA analysis (VIP > 1.0 and p < 0.05).

Serial Number	Compounds	Class I	VIP	*P*-value	Fold Change	Log_2_FC	Type
1	Benzenemethanol, .alpha.-(trichloromethyl)-, acetate	Ester	1.05	0.0016	1626.76	10.67	up
2	4aH-Cycloprop[*e*]azulen-4a-ol, decahydro-1,1,4,7-tetramethyl-, [1aR-(1a.alpha.,4.beta.,4a.beta.,7.alpha.,7a.beta.,7b.alpha.)]-	Terpenoids	1.05	0.0025	1301.90	10.35	up
3	Ledol	Terpenoids	1.05	0.0025	1301.90	10.35	up
4	Butanoic acid, 2-methyl-, 3,7-dimethyl-2,6-octadienyl ester, (*Z*)-	Ester	1.05	0.0025	1258.37	10.30	up
5	Butanoic acid, 3-methyl-, 3,7-dimethyl-2,6-octadienyl ester, (Z)-	Acid	1.05	0.0025	1250.43	10.29	up
6	Butanoic acid, 2-methyl-, 3,7-dimethyl-2,6-octadienyl ester, (*E*)-	Ester	1.05	0.0025	1250.43	10.29	up
7	Geranyl isovalerate	Ester	1.05	0.0025	1250.43	10.29	up
8	Benzoic acid, 3-nitro-	Ester	1.05	0.0025	1256.12	10.29	up
9	1,6,10-Dodecatrien-3-ol, 3,7,11-trimethyl-, (E)-	Ester	1.05	0.0022	1114.16	10.12	up
10	Nerolidol	Terpenoids	1.05	0.0022	1114.16	10.12	up
11	Cis-Nerolidol	Terpenoids	1.05	0.0022	1114.16	10.12	up
12	Benzocaine	Terpenoids	1.05	0.0023	1126.14	10.14	up
13	Caryophyllene oxide	Terpenoids	1.05	0.0023	1088.49	10.09	up
14	2,4,4-Trimethyl-3-(3-methylbutyl)cyclohex-2-enone	Ketone	1.05	0.0024	1002.44	9.97	up
15	3-Amino-4-pyrazolecarbonitrile	Heterocyclic compound	1.05	0.0023	854.11	9.74	up

Data shows variable importance in projection (VIP) scores, *p*-values, fold changes, and log2 fold changes for significantly different compounds (*p* < 0.01).

**Table 2:** All significantly down-regulated volatile compounds in ‘Fenyu’ (FY) compared to wild ‘HM’ strawberries, identified by PLS-DA analysis (VIP > 1.0 and p < 0.05).

Serial Number	Compounds	Class I	VIP	P-value	Fold Change	Log_2_FC	Type
1	Geranyl acetate	Terpenoids	1.05	0.0006	0.0006	−10.59	down
2	Benzene, (1-methyl-1-propylpentyl)-	Aromatics	1.05	0.0002	0.0010	−9.98	down
3	trans-O-Dithiane-4,5-diol	Alcohol	1.05	0.0001	0.0012	−9.65	down
4	Ethyl mandelate	Ester	1.05	0.0007	0.0026	−8.57	down
5	Benzoic acid, ethyl ester	Ester	1.05	0.0000	0.0046	−7.77	down
6	4-Hexen-1-ol, (E)-	Alcohol	1.05	0.0001	0.0053	−7.55	down
7	4-Hexen-1-ol, (Z)-	Alcohol	1.05	0.0001	0.0053	−7.55	down
8	3-Pyridinecarboxamide, *N*-methyl-	Heterocyclic compound	1.05	0.0002	0.0064	−7.28	down
9	Diisopropyl adipate	Ester	1.04	0.0006	0.0084	−6.90	down
10	Benzaldehyde, 3,5-dimethyl-	Aldehyde	1.03	0.0000	0.0085	−6.88	down
11	1,3-Dioxepane, 2-heptyl-	Heterocyclic compound	1.05	0.0002	0.0095	−6.71	down
12	2-Butenal, 3-methyl-	Aldehyde	1.05	0.0003	0.0101	−6.63	down
13	3-Phenyl-1-propanol, acetate	Ester	1.05	0.0002	0.0116	−6.43	down
14	3-Acetoxy-2-butanone	Ketone	1.05	0.0087	0.0118	−6.40	down
15	2,3-Dimethyl-5-n-propylpyrazine	Heterocyclic compound	1.05	0.0001	0.02	−6.00	down

Data shows variable importance in projection (VIP) scores, p-values, fold changes, and log2 fold changes for significantly different compounds (p < 0.01).

The analysis of volatile compounds in the cultivated strawberry variety FY compared to the wild variety ‘HM’ revealed significant downregulation of key aroma-related metabolites (Table 2). The most pronounced reduction was observed for geranyl acetate, a terpenoid associated with floral and citrus notes, which exhibited a striking log2FC of −10.59, indicating a near-complete suppression in FY relative to HM (Table 2). Similarly, aromatic and ester compounds such as benzene derivatives and ethyl mandelate showed substantial downregulation, with log2FC values ranging from −9.98 to −7.77 (Table 2). The dramatic reduction in these compounds suggests a potential trade-off, where breeding for enhanced sweetness or yield in cultivated varieties may have come at the expense of complex floral and aromatic notes retained in wild genotypes.

The downregulated compounds include diverse chemical classes such as alcohols, aldehydes, and heterocyclic compounds, further underscoring the broad metabolic shifts associated with interspecific differences (Table 2). For instance, trans-O-Dithiane-4,5-diol and 4-hexen-1-ol isomers, both alcohols contributing to green and fresh aroma notes, were significantly reduced (log2FC -9.65 and − 7.55, respectively) (Table 2). Esters like diisopropyl adipate and 3-phenyl-1-propanol acetate, which contribute to fruity and sweet aromas, also showed notable declines (log2FC -6.90 and − 6.43) (Table 2). Overall, the findings provide actionable insights into breeding programs, suggesting that reintroducing or modulating these suppressed pathways could enhance the complexity and diversity of aroma profiles in commercial strawberries while potentially restoring lost defensive or stress-responsive traits.

### 3.6 Correlation heatmap of MYB Gene-volatile compound

**Fig. 5:**
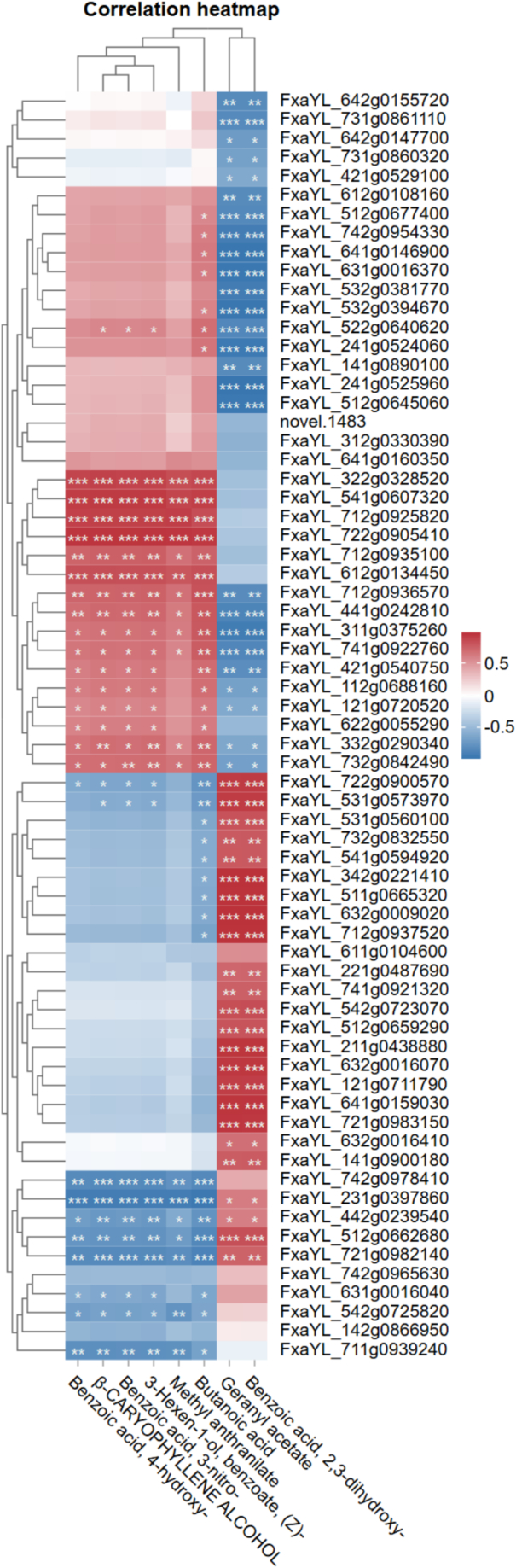
Correlation network between MYB genes (genes used for correlation are represented in supplementary table S4) and volatiles (compounds used for correlation are represented in supplementary table S5) in strawberry varieties. Heatmap displays Pearson correlation coefficients (red: positive; blue: negative) between MYB gene expression levels and volatile metabolite abundances across wild (HM) and cultivated (DX, FY, RF) strawberries. Gray indicates nonsignificant correlations (|r| < 0.5, *p > 0.05, **p > 0.01, ***p > 0.001). (For interpretation of the references to color in this figure legend, the reader is referred to the web version of this article.)

To establish robust regulatory relationships, correlation analysis was performed specifically between the expression of differentially expressed MYB genes and the abundance of volatile compounds that were significantly altered (VIP > 1.0 and |log2FC| ≥ 1) between the wild HM and the pooled cultivated varieties (Supplementary Table S3). The correlation heatmap analysis revealed significant associations between MYB transcription factors and volatile organic compounds (VOCs) across four strawberry varieties (Fig. 5). Strong positive correlations (red clusters, ***p < 0.001) were identified between specific MYB genes (FxaYL_542g0723070, FxaYL_512g0659290, FxaYL_211g0438880, FxaYL_632g0016070, FxaYL_121g0711790, FxaYL_641g0159030, and FxaYL_721g0983150) and key aroma compounds including 2,3-dihydroxy-benzoic acid and geranyl acetate (Fig. 5). These strong associations suggest these MYB genes may function as positive regulators in the biosynthesis pathways of these important flavor and fragrance compounds, which contribute to the characteristic fruity and floral notes of strawberry.

A second distinct cluster of MYB genes (FxaYL_322g0328520, FxaYL_541g0607320, FxaYL_712g0925820, and FxaYL_722g0905410) showed significant positive correlations with a different set of aroma compounds including butanoic acid, methyl anthranilate, 4-hydroxy-benzoic acid, benzoate (Z)-3-Hexen-1-ol, β-caryophyllene alcohol, and 3-nitro-benzoic acid (Fig. 5). This finding suggests these MYBs are associated with multiple branches of the volatile biosynthesis network. In contrast, a small subset of MYB genes (FxaYL_742g0978410, FxaYL_231g0397860, and FxaYL_721g0982140) exhibited strong negative correlations with these same compounds, potentially indicating their role as repressors in these metabolic pathways. These opposing regulatory patterns underscore a complex transcriptional balance, wherein specific MYBs appear to coordinate through both activation and suppression to shape the strawberry volatile profile.

The strong correlations between MYB genes and key aroma compounds provide valuable insights into potential regulatory networks, suggesting these transcription factors may modulate volatile biosynthesis by influencing the expression of biosynthetic enzymes. For instance, the association of multiple MYBs with geranyl acetate aligns with known MYB functions in terpenoid regulation in other species. We emphasize that these proposed roles for the identified MYBs are hypotheses based on their correlated expression with metabolites, pending confirmation through phylogenetic analysis and functional validation. Future studies to functionally characterize these candidates are essential to define their precise roles and will provide a robust foundation for molecular breeding strategies aimed at enhancing strawberry aroma.

## 4 Discussion

The findings of this study showed that breeding and selection have significantly reshaped the VOC profiles and MYB transcription factor expression patterns in strawberries, aligning with previous research on the genetic and metabolic divergence between wild and cultivated varieties (Chamorro et al., 2025). The hierarchical clustering and PCA analyses revealed distinct separation between wild (*Fragaria nilgerrensis* ‘HM’) and cultivated (*F. × ananassa* ‘Red Face 99’, ‘Fenyu’, and ‘Danxue’) strawberries, with the latter exhibiting elevated levels of fruity and floral VOCs such as nerolidol, methyl anthranilate, and esters (Fig. 3). These results corroborate earlier studies demonstrating that human selection often favors enhanced sensory attributes, such as sweetness and aroma complexity, at the expense of defensive compounds retained in wild relatives (Fan & Whitaker, 2024). For instance, the upregulation of benzenemethanol acetate and geranyl isovalerate in cultivated varieties mirrors findings in tomatoes and grapes, where breeding has favored esters and terpenoids associated with consumer appeal (Distefano et al., 2022). Conversely, the wild ‘HM’ genotype retained higher levels of geranyl acetate and 2,3-dihydroxy-benzoic acid, compounds linked to stress resistance and herbivore deterrence, underscoring the trade-off between palatability and ecological adaptation (Chamorro et al., 2025; Wang et al., 2024). This metabolic divergence highlights the potential of reintroducing wild-type traits into cultivated varieties to balance flavor and resilience. However, a limitation of this study is its focus on a single wild accession. Expanding this work to include diverse *F. nilgerrensis* germplasm will be important to generalize these results and pinpoint the most valuable wild traits for improvement of cultivated varieties.

The GC–MS metabolomic data revealed striking differences in VOC abundance among strawberry varieties, with cultivated types exhibiting higher levels of compounds such as nerolidol and hexanoic acid methyl ester, which contribute to floral and fruity notes (Fig. 2, Fig. 3, Fig. 4). These results are consistent with sensory evaluations of commercial strawberries, where esters and terpenoids are key determinants of consumer preference (He et al., 2025; Zheng et al., 2025). Notably, the wild ‘HM’ genotype displayed a distinct VOC profile dominated by geranyl acetate and ethyl mandelate, compounds often associated with defensive functions. These parallels findings in wild tomatoes and grapes, where ecological pressures maintain higher levels of phenolics and volatile aldehydes (Gascuel et al., 2017). The downregulation of these compounds in cultivated strawberries suggests a metabolic trade-off, wherein breeding for sweetness and yield may inadvertently diminish aromatic complexity. However, the retention of some wild-type VOCs in certain cultivars, such as ‘Fenyu’, hints at the potential for introgression of resilience-linked traits without compromising flavor. Such strategies have been successfully employed in wine grapes, where hybrid varieties combine disease resistance with desirable aroma profiles (González-Centeno et al., 2019).

The role of MYB transcription factors in regulating strawberry aroma pathways emerged as a central theme in this study, with specific MYB genes showing strong correlations with key VOCs. For example, FxaYL_542g0723070 and FxaYL_512g0659290 were positively associated with 2,3-dihydroxy-benzoic acid and geranyl acetate, suggesting their involvement in phenylpropanoid and terpenoid biosynthesis. These findings align with prior work in blueberries and *Dendrobium officinale*, where MYB genes were shown to modulate anthocyanin and monoterpene production (Lv et al., 2022; Plunkett et al., 2018; M. Zhao et al., 2019). Similarly, the negative correlation between FxaYL_742g0978410 and fruity esters implies a repressive role, consistent with reports of MYBs acting as both activators and suppressors of secondary metabolism (Rao & Zheng, 2025; Tak et al., 2017). The differential expression of MYB genes between wild and cultivated varieties showed that breeding and selection has selectively amplified regulatory networks favoring desirable flavor traits (Aharoni et al., 2004). This is analogous to the upregulation of MYB genes in domesticated citrus and peaches, which is associated with the accumulation of secondary metabolites, volatile esters, and lactones (Li et al., 2023; Rao et al., 2021). Collectively, these insights position MYBs as prime targets for precision breeding aimed at enhancing aroma complexity while preserving agronomic robustness (Jian et al., 2019).

The prospect of leveraging MYB transcription factors for breeding is further illuminated by emerging models of sophisticated regulatory hierarchies in strawberries. A paradigm for such complexity is the antagonistic relationship between FaMYB44.2, a transcriptional repressor of sucrose accumulation, and FaMYB10, a master regulator of anthocyanin biosynthesis (Wei et al., 2018; Yue et al., 2023). In this established module, FaMYB10 directly inhibits *FaMYB44.2* expression, thereby de-repressing sucrose biosynthesis to coordinate ripening-associated traits (Wei et al., 2018). We propose that an analogous regulatory conflict may underpin the flavor-defense trade-off observed in our study. Specifically, the wild genotype-enriched MYBs correlating with defense compounds (e.g., FxaYL_632g0009020; Fig. 1A) could function as repressors of fruity volatile biosynthesis, similar to FaMYB44.2's role in sugar metabolism (Wei et al., 2018). Conversely, modern breeding may have favored selection for activator MYBs (e.g., FxaYL_642g0147700) that suppress these repressive counterparts, leading to the concurrent enhancement of sugar and aroma volatile production, a potentially co-regulated “sweet aroma” regulon. This hypothesis offers a testable molecular framework for strawberry domestication, positing that selection acted upon master regulatory switches capable of reprogramming integrated metabolic networks.

The correlation heatmap analysis revealed significant associations between specific MYB genes and key aroma compounds. For instance, the positive association between FxaYL_712g0925820 and methyl anthranilate is consistent with findings in *Medicago truncatula*, where MYBs regulate this compound's production, suggesting a potentially conserved regulatory role (Pollier et al., 2019). Similarly, the negative correlation between FxaYL_231g0397860 and β-caryophyllene alcohol suggests a repressive role, similar to R2R3-MYB-mediated suppression of monoterpene in spearmint (Reddy et al., 2017). These regulatory patterns underscore the complexity of strawberry aroma biosynthesis, where MYBs act as master switches coordinating multiple metabolic pathways. The study's integrated approach (combining transcriptomics and metabolomics) mirrors recent advances in fruit flavor research, such as the dissection of melon aroma networks (Kaleem et al., 2024). By identifying candidate MYB genes for targeted manipulation, this work lays the groundwork for breeding strawberries with enhanced sensory profiles, akin to efforts in Arabidopsis, citrus, and apple fruits (D. Zhao et al., 2021). While this correlation-based approach identifies compelling candidate MYBs associated with aroma or defense, future studies involving phylogenetic comparison with reference species (e.g., Arabidopsis) and functional validation (e.g., through gene knockout or overexpression) will be essential to definitively confirm their specific roles in strawberry volatile biosynthesis.

## 5 Conclusion

This study provides compelling evidence for a significant divergence in the volatile profiles and regulatory networks between wild and cultivated strawberries, with cultivated varieties favoring fruity and floral aroma compounds while wild genotypes retain metabolites linked to stress adaptation. The distinct clustering of MYB gene expression and VOC signatures between wild (*F. nilgerrensis* ‘HM’) and cultivated (*F. × ananassa*) strawberries underscores the genetic trade-offs between flavor quality and ecological resilience. Key findings highlight the role of specific MYB transcription factors such as FxaYL_542g0723070 and FxaYL_512g0659290, in modulating desirable aroma compounds like nerolidol and methyl anthranilate, offering promising targets for molecular breeding. The downregulation of defensive volatiles (e.g., geranyl acetate) in cultivated varieties suggests opportunities to reintroduce wild-type traits without compromising sensory appeal. By integrating metabolomic and transcriptomic approaches, this work advances our understanding of strawberry flavor biochemistry and provides a foundation for developing improved cultivars with enhanced aroma complexity and agronomic robustness. Future research should focus on functional validation of candidate MYB genes and their application in precision breeding strategies for *Fragaria* and other fruit crops.

## CRediT authorship contribution statement

**Mingzheng Duan:** Writing – review & editing, Writing – original draft, Software, Resources, Methodology, Investigation, Funding acquisition. **Kangjian Song:** Writing – review & editing, Investigation. **Ting Jiang:** Writing – review & editing, Investigation. **Xiaoting Fu:** Writing – review & editing, Investigation. **Huaming Lei:** Writing – review & editing, Investigation. **Jieming Feng:** Writing – review & editing, Investigation. **Congjing Chen:** Writing – review & editing, Investigation. **Xiande Duan:** Writing – review & editing, Investigation. **Shunqiang Yang:** Writing – review & editing, Resources, Funding acquisition, Conceptualization. **Muhammad Junaid Rao:** Writing – review & editing, Writing – original draft, Software, Methodology, Conceptualization.

## Funding

This work was jointly funded by the Academician and Expert Workstation of Yunnan Province. Grant number: 202305AF150183; The project of Scientific research start-up funds for doctoral talents of Zhaotong University - Mingzheng Duan, Grant number: 202406; Young Talent Project of Talent Support Program for the Development of Yunnan. Grant number: 210604199008271015; Team Project of the “Xingzhao Talent Support Plan” in Zhaotong City. Grant No.: ZhaodangRencai[2023]No.3.

## Declaration of competing interest

The authors declare that they have no known competing financial interests or personal relationships that could have appeared to influence the work reported in this paper.

## Data Availability

Data will be made available on request.

## Appendix A Supplementary data

Supplementary material 1 OPLS-DA scores and S-plot of volatile organic compounds from four strawberry varieties. Image 1

Supplementary material 2 Raw transcriptomic and metabolomic data. Image 2
