# Supplementary material for: Divergent volatilomes between wild and cultivated strawberries: MYB transcription factors underlie flavor differences
Source: Food Chem (Oxf). 2025 Nov 4;11:100323. doi: 10.1016/j.fochms.2025.100323 (PMC12648578; doi:10.1016/j.fochms.2025.100323)
Supplement: Supplementary material 1 — OPLS-DA scores and S-plot of volatile organic compounds from four strawberry varieties. [file mmc1.docx]

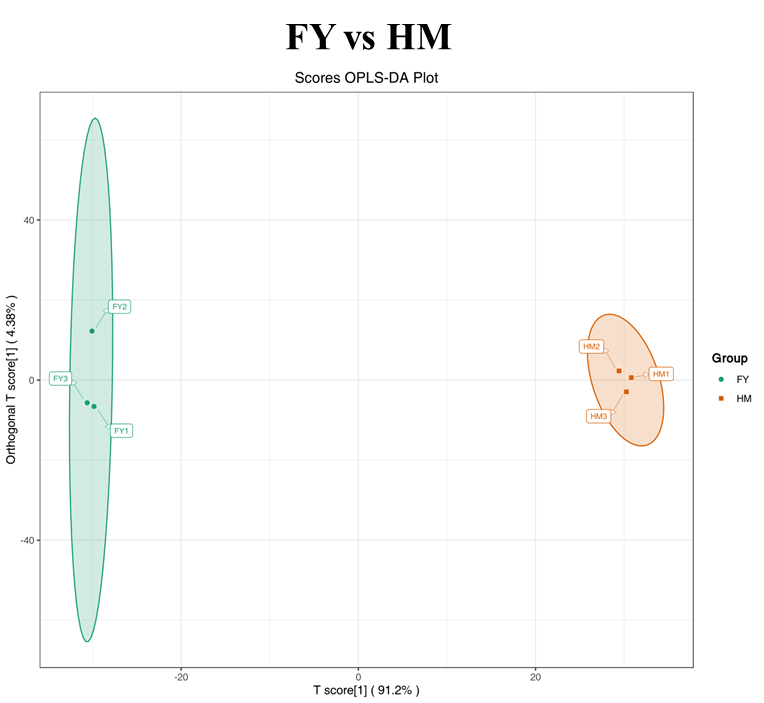

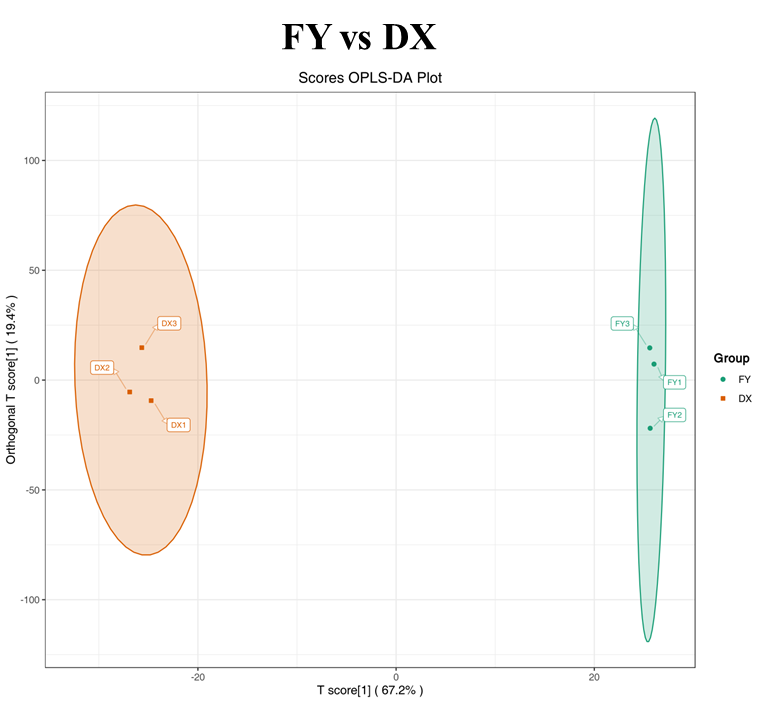

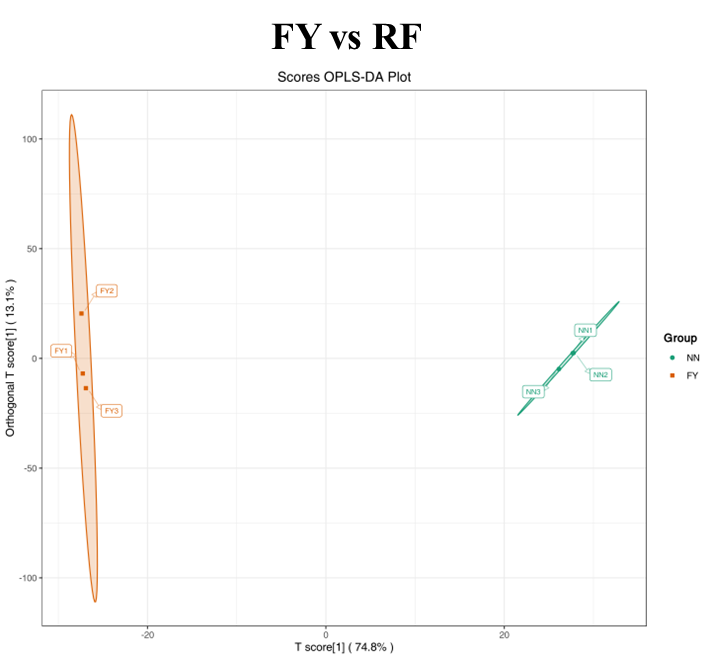

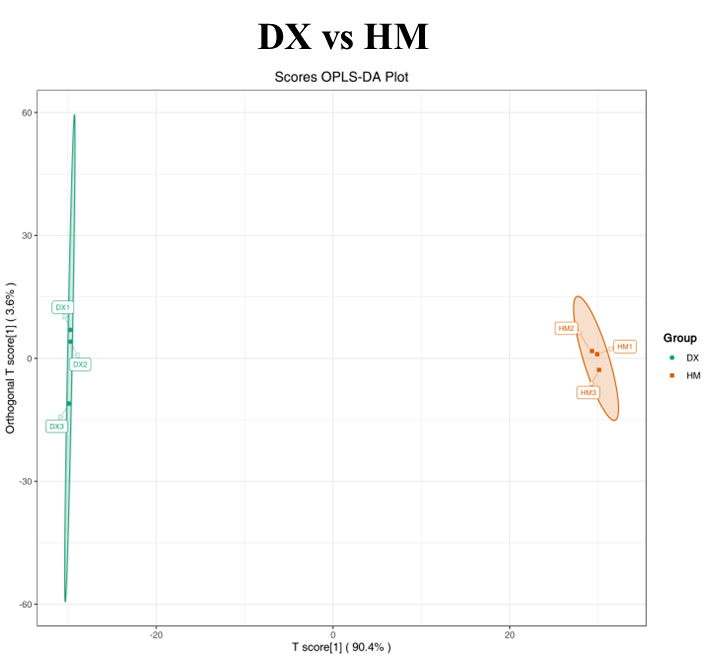

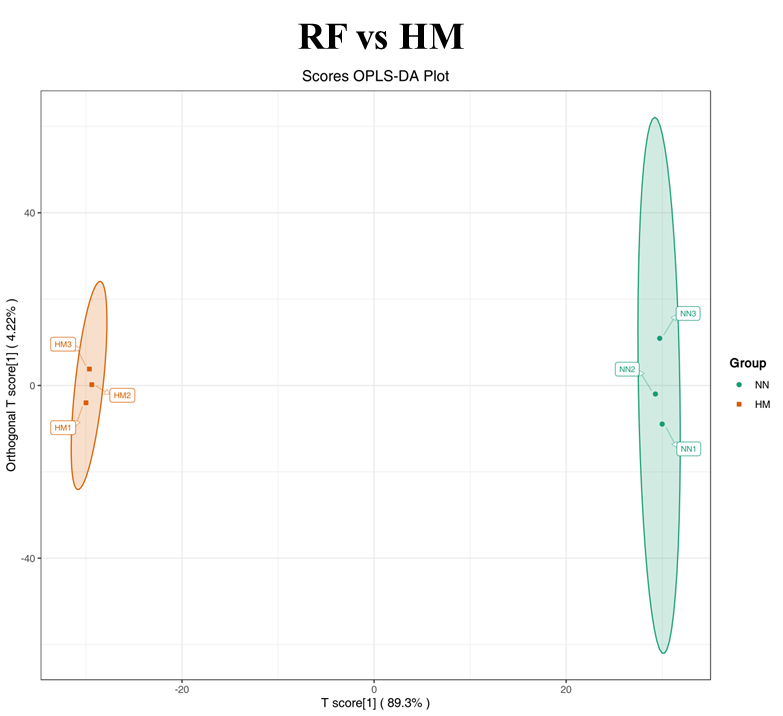


**Supplementary Figure S1.** OPLS-DA scores plot of volatile organic compounds from four strawberry varieties. The plot displays the separation between the sample groups (HM, DX, FY, RF) based on their volatile profiles (NN or RF are same). The X-axis (t[1]) represents the predictive component that captures the variation between groups. The Y-axis (t[2]) represents the orthogonal component that captures the variation within groups. The percentage values indicate the variance explained by each component. Each point represents an individual biological replicate, and ellipses represent the 95% confidence interval for each group.


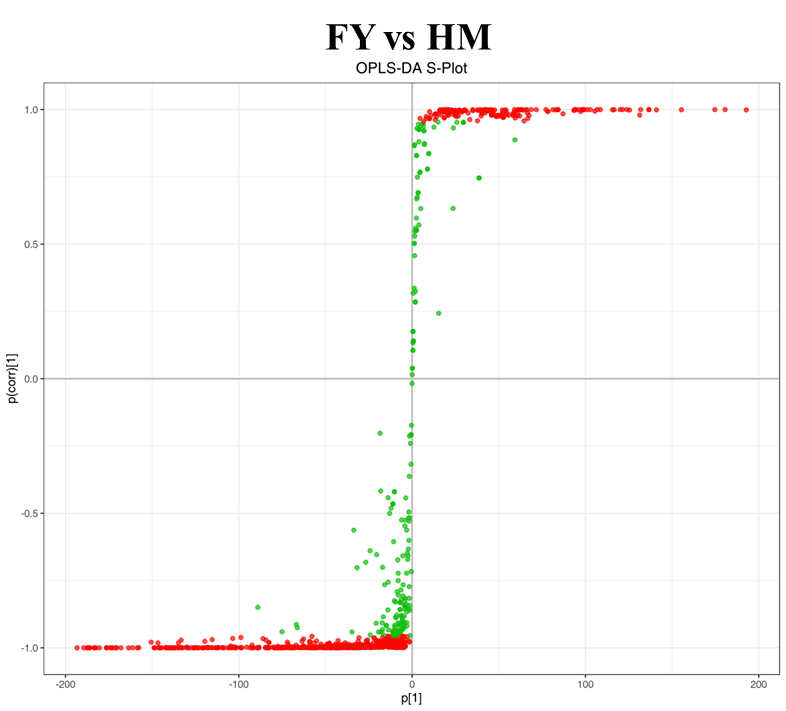

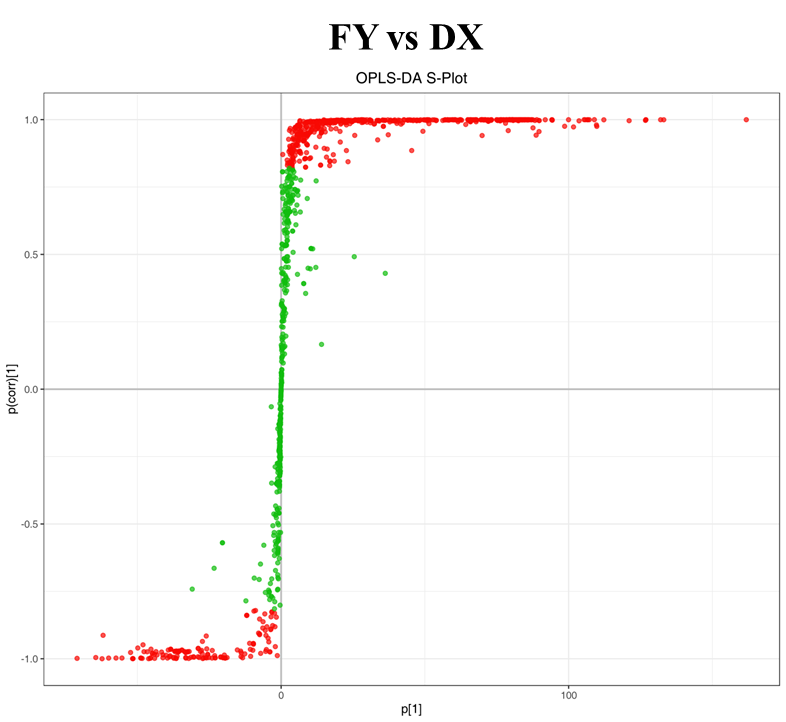

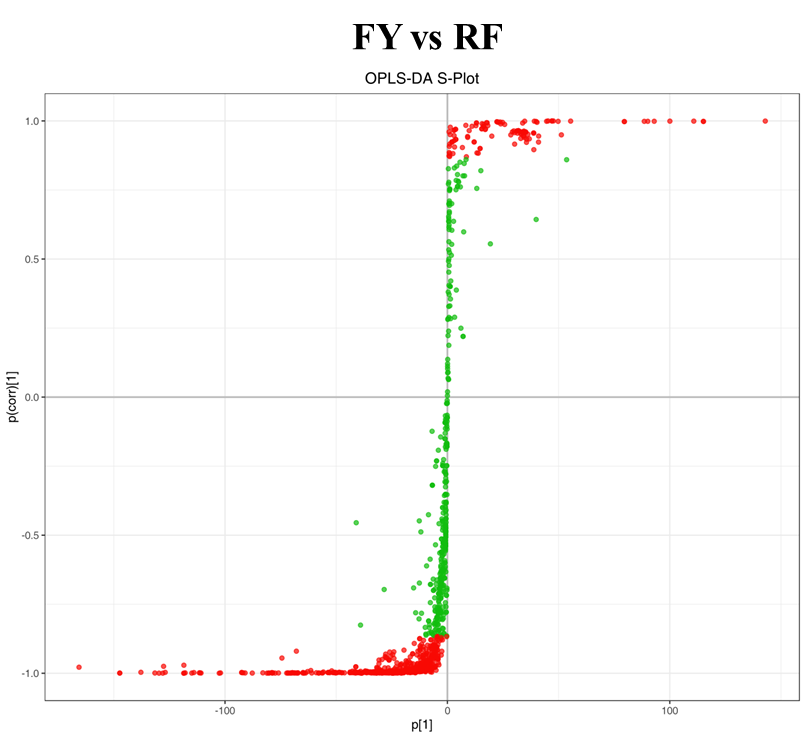

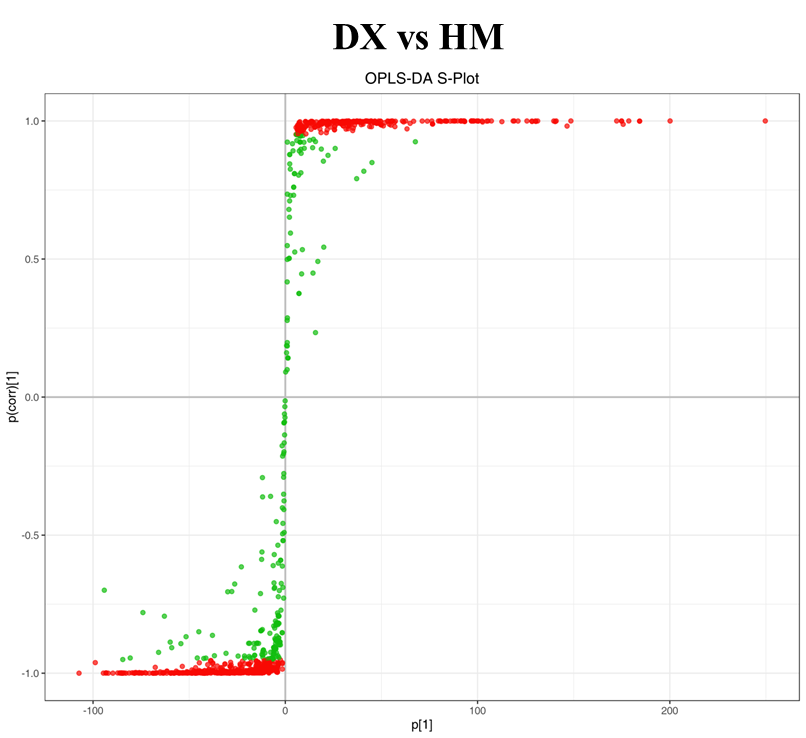

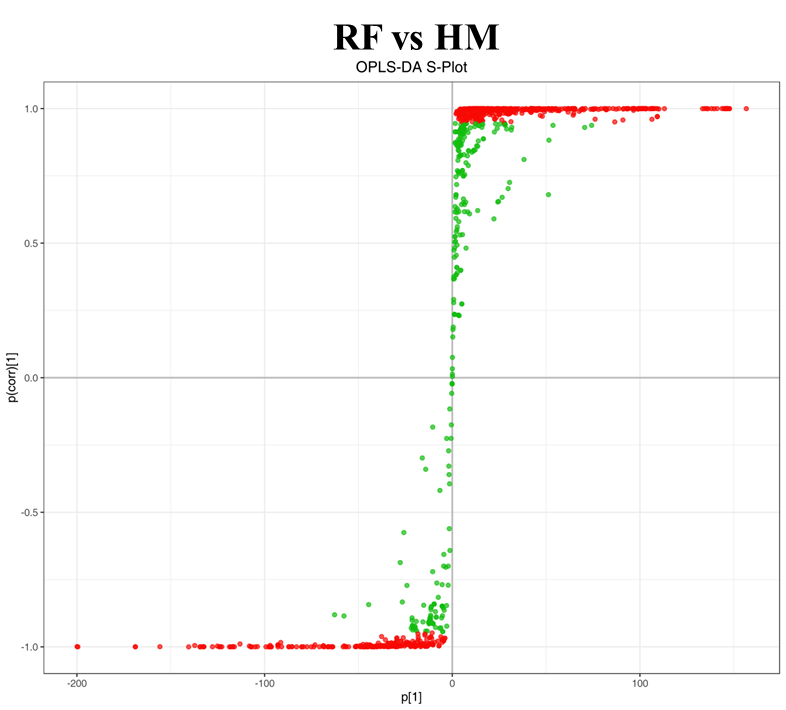


**Supplementary Figure S2.** S-plot from the OPLS-DA model of strawberry volatile profiles. The S-plot visualizes the contribution and reliability of metabolites to the group separation observed in the OPLS-DA scores plot. The X-axis (p[1]) represents the covariance, indicating the contribution magnitude of each metabolite. The Y-axis (p(corr)[1]) represents the correlation, indicating the reliability of the contribution. Metabolites furthest from the origin in the top-right and bottom-left corners have the highest combination of contribution and reliability and are thus the most significant biomarkers. Metabolites with a VIP value > 1 are highlighted in red, while those with VIP ≤ 1 are shown in green.
